# Plant-endophyte mediated improvement in physiological and bio-protective abilities of marigold (*Tagetes patula*)

**DOI:** 10.3389/fpls.2022.993130

**Published:** 2022-09-09

**Authors:** Muhammad Naveed, Sidra Hafeez, Munazza Rafique, Muhammad Zahid Mumtaz, Zinayyera Subhani, Jiri Holatko, Tereza Hammerschmiedt, Ondrej Malicek, Adnan Mustafa, Antonin Kintl, Martin Brtnicky

**Affiliations:** ^1^Institute of Soil and Environmental Sciences, University of Agriculture, Faisalabad, Pakistan; ^2^Soil Bacteriology Section, Ayub Agricultural Research Institute, Faisalabad, Pakistan; ^3^Institute of Molecular Biology and Biotechnology, The University of Lahore, Lahore, Pakistan; ^4^Faculty of Life Sciences, University of Central Punjab, Lahore, Pakistan; ^5^Agrovyzkum Rapotin Ltd., Rapotin, Czechia; ^6^Department of Agrochemistry, Soil Science, Microbiology and Plant Nutrition, Faculty of AgriSciences, Mendel University in Brno, Brno, Czechia; ^7^Institute of Chemistry and Technology of Environmental Protection, Faculty of Chemistry, Brno University of Technology, Brno, Czechia; ^8^Institute for Environmental Studies, Faculty of Science, Charles University, Prague, Czechia; ^9^Agricultural Research, Ltd., Troubsko, Czechia

**Keywords:** allelopathic effect, antioxidant activity, endophytic bacteria, metabolites, plant-parasitic nematodes, *Tagetes patula*, vase life

## Abstract

Endophytic bacteria improve the growth, physiology, and metabolite profile of plants. They are known as potential biocontrol agents of soil-borne diseases. This study evaluated the effects of endophytic bacterial strains on growth, vase life, biochemical attributes, and antioxidant and nematicidal activities of French marigold (*Tagetes patula*). French marigold seeds were sole and consortium inoculated with three promising endophytic bacterial strains, *Burkholderia phytofirmans* (PsJN), *Enterobacter* sp. (MN17), and *Bacillus* sp. (MN54). The vase life of French marigold was promoted by 66.6% in the individual application of PsJN and 100% in plants treated with consortium compared to the uninoculated control. The shoot and root fresh weights were also increased by 65.9 and 68.7%, with the combined application of all three strains. The total phenolics, flavonoid, and protein contents were higher in consortium treatment with an increase of up to 38.0, 55.9, and 65.9%, respectively, compared to the uninoculated control. Furthermore, combined application of endophytic bacterial strains promoted DPPH radical scavenging, mortality of plant-parasitic nematodes, and ferric reducing antioxidant power activities with increase of up to 278.0, 103.8, and 178.0%, respectively, compared to uninoculated control. An increase in antioxidant activities of ascorbate peroxidase (APX), catalase (CAT), glutathione peroxidase (GPX), and superoxide dismutase (SOD) were observed up to 77.3, 86.0, 91.6, and 102.9%, respectively by combined application of endophytic bacterial strains. So, given the economic importance of floriculture crops, endophytic bacterial isolates studied here have shown a great potential for improving the productivity of cultivated ornamental French marigold.

## Introduction

Floriculture contributes excellent value to the agricultural economy of Pakistan. Significant growth has been observed in this sector resulting in massive production of ornamental plants (Sudhagar, 2013; Khan et al., 2016). French marigold (*Tagetes patula*) is a commercial ornamental flower used in medicinal products for decoration and landscaping purposes (Bosma et al., 2003). It is also a repellent plant against various Lepidoptera, Coleoptera, Hemiptera, and plant-parasitic nematodes (PPNs; Fabrick et al., 2020). French marigold is a widely studied plant due to its allelopathic potential against PPNs (Hooks et al., 2010). Literature indicated that marigolds could be involved in the biocontrol of 14 genera of PPNs, including ectoparasitic, endoparasitic, and semi-endoparasitic nematodes (Suatmadji, 1969; Siddiqui and Alam, 1987). Marigold affects PPNs by acting as a poor host, producing allelopathic compounds, creating an antagonistic nematode environment, and behaving as a trap crop (Wang et al., 2001; Pudasaini et al., 2008). The expansion of environmentally safe techniques to inhibit PPNs growth seems attractive due to the environmental hazards of nematicides and chemical fumigants (Schneider et al., 2003).

The commercial production of ornamental plants is significant globally, including in Pakistan. Essential plant nutrients are vital in the quality production of seeds and flowers (Kashif, 2001). Over the past decades, extensive use of chemical fertilizers has negatively impacted the environment. In this regard, the application of bioinoculants has been gaining interest in the scientific communities (Zahir et al., 2004; Khan et al., 2019; Nazli et al., 2020). Several plant growth-promoting rhizobacteria (PGPR) have shown their potential to improve plant growth, crop yield, and quality (Ali et al., 2017; Mustafa et al., 2019; Umar et al., 2020). Such bioinoculants have direct effects on plant growth as these species facilitate nutrient uptake, fix atmospheric nitrogen (N), solubilize nutrients including phosphorus (P), potassium (K), and zinc (Zn), produce siderophores which solubilize and sequester iron, synthesize growth hormones (e.g., auxins, cytokinins, and gibberellins), and synthesize the enzymes that modulate plant growth and development (Gray and Smith, 2005; Khan and Bano, 2019; Saeed et al., 2019). These microbes enhance plant growth indirectly by reducing or eliminating the adverse effects of pathogenic microorganisms using numerous mechanisms that include the induction of host resistance to the pathogen (Van Loon, 2007; Khan, 2020; Khan et al., 2020; Nazli et al., 2020). Multiple studies have demonstrated the improvement in plant growth and development following seed or root inoculation with microbial strains capable of producing plant growth regulators (Zahir et al., 2004; Naseem et al., 2018; Ahmad et al., 2019b).

Certain aspects of PGPR interactions have been studied well, e.g., growth effects, nutrient availability, biocontrol of plant pathogens, tolerance of water stress, and other adverse environmental conditions. The microbiota within plant roots may significantly differ from that within the rhizosphere, indicating that the plants influence the microbial colonies inside their roots (Ali et al., 2017). Some endophytes can promote plant growth, and the mechanisms adopted by bacterial endophytes are similar to those used by rhizospheric bacteria (Santoyo et al., 2016). Within their plant host, the microbes named endophytes remain in the intercellular or intracellular region of healthy plant tissue throughout their complete life cycle, causing no harmful impact on the plant. By secreting phytohormones, endophytic bacteria promote the growth of their host. Endophytic bacteria can also promote the growth and yield of a plant by acting as biocontrol agents (Mustafa et al., 2019).

Flower senescence is the leading cause of short vase life and loss in quality which primarily determines its economic and ornamental value to establish a capital-incentive business (Van Doorn, 2002). Ethylene is a crucial stress regulatory phytohormone produced at low levels under normal circumstances and conferring beneficial effects on plant growth and development (Ahmad et al., 2020). However, in response to various stresses, there is often a significant increase in endogenous ethylene production that leaves adverse impacts on plant growth and is thought to be responsible for flower senescence (Van Doorn, 2001). Endophytic microbes having ACC-deaminase activity may naturally enhance plant growth under stress conditions by lowering ethylene production (Nascimento et al., 2012; Nazli et al., 2020). Flower senescence can be delayed by a reduction in ACC content, causing the synthesis of ethylene to a smaller extent (Moon and Ali, 2022). Previously, several studies have reported positive effects of PGPR and endophytic inoculation on plant growth promotion of different crops. However, the impact of microbial consortium inoculation remained neglected in the past, especially in horticultural crops. Moreover, there is a need to explore the effects of microbial inoculation on pharmacological and nematicidal activities of crops such as French marigold, which in the present study constitutes the further novelty of this work. Thus, we hypothesized that the endophytic bacterial inoculation in consortia might improve the growth and extends the vase life of French marigold; however, their effects on pharmacological and nematicidal activities may vary depending on the microbial sp. in question. The specific objectives of the present study were to evaluate the impact of different endophytic bacterial species on growth, flowering, and delay in flower senescence in French marigolds and the effects of bacterial endophytes on pharmacological and nematicidal activities of essential flower extract of French marigolds.

## Results

### Plant growth parameters

The present study shows the effects of endophytic bacteria strain *Burkholderia phytofirmans* PsJN, *Enterobacter* sp. MN17, and *Bacillus* sp. MN54 inoculation on French marigold growth and senescence. Almost all the growth parameters evaluated were significantly (*p* < 0.05) modified by four inoculating treatments.

**FIGURE 1:**
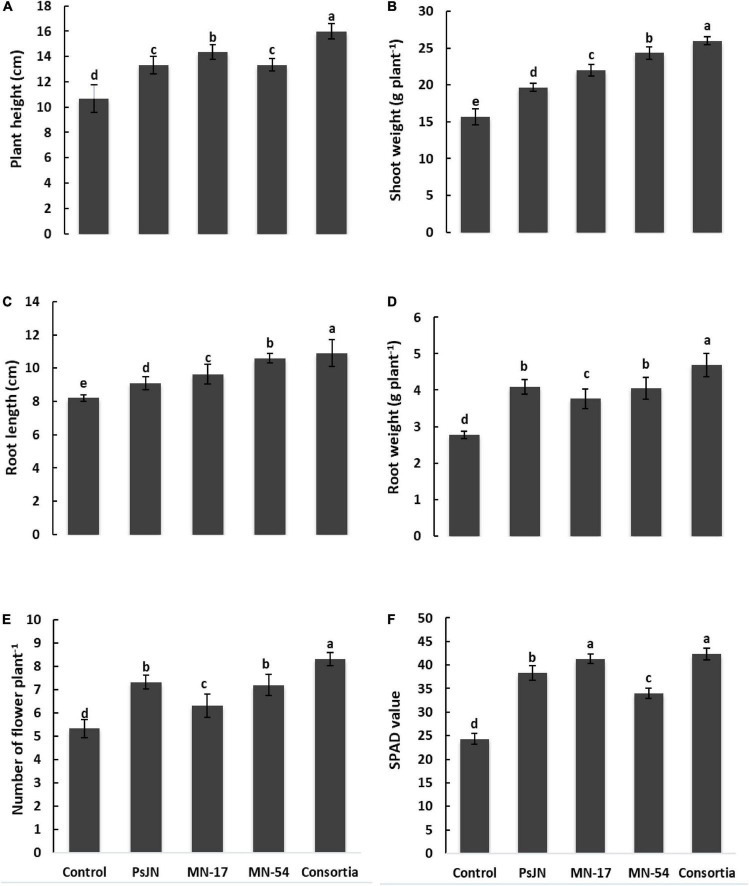
Effect of endophytic bacterial inoculation on **(A)** plant height, **(B)** shoot weight, **(C)** root length, **(D)** root weight, **(E)** number of flowers plant^–1^, and **(F)** SPAD value of French marigold. Error bars represent the standard error (SE). Bars with different letters are significantly different (*P* < 0.05) according to the LSD test.

Inoculation with endophytic bacteria caused a significant increase in plant height, shoot mass, root mass, and root length (Figure 1). All the five treatments statistically differed from each other. The increase in plant height up to 15.98 cm was recorded in the consortia, followed by MN17 showed an increase in plant height (14.33 cm) which was significantly different from control. The minimum plant height of 10.67 cm was found in control. The difference in plant height in PsJN, MN17, MN54, and consortia was quite evident compared to control. Data regarding shoot fresh weight revealed that application of endophytic bacterial strains significantly improved the shoot fresh weight plant^–1^. The maximum increase in shoot fresh weight up to 26 g plant^–1^ was recorded in the consortia application, followed by MN54, MN17, and PsJN showed an increase in shoot fresh weight up to 24.33, 22.00, and 19.68 g plant^–1^, respectively, compared to control (15.67 g plant^–1^; Figure 1).

Inoculation with MN54 significantly affects root length (10.60 cm) and root fresh weight (4.05 g plant^–1^), which was further increased in consortia application (10.90 cm and 4.69 g plant^–1^, respectively) as compared to control (8.2 cm and 2.78 g plant^–1^, respectively; Figure 1). Individual inoculation of PsJN and consortia significantly increased the number of flowers plant^–1^ up to 7.33 and 8.33, respectively, over control which reported 5.30 number of flowers plant^–1^.

### Endophytic bacteria extend the vase life

**FIGURE 2:**
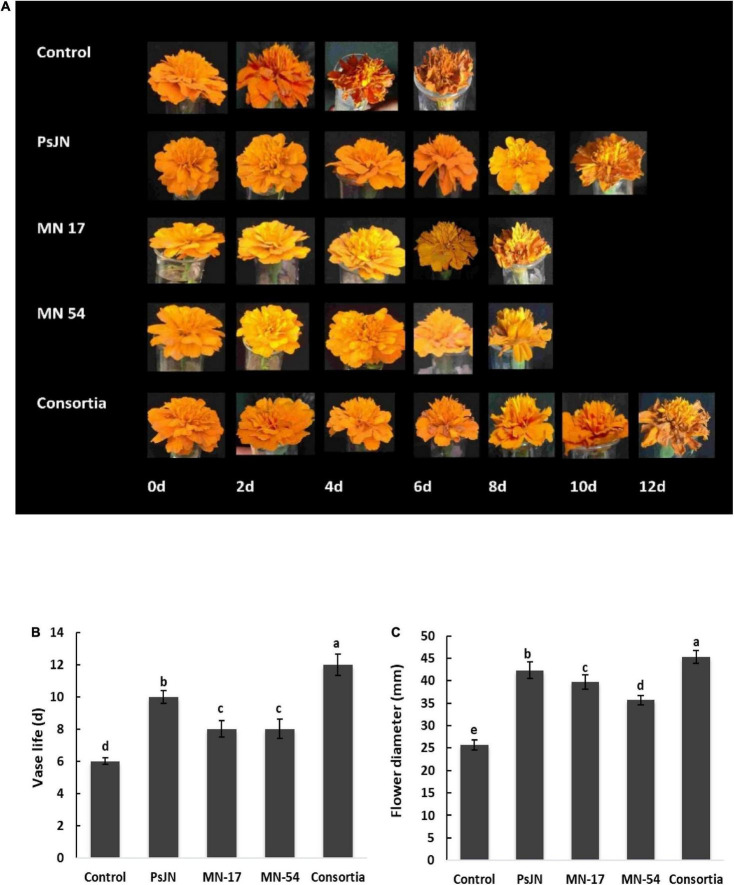
Effect of endophytic bacterial inoculation on **(A,B)** vase life and **(C)** maximum flower diameter of French marigold. Error bars represent the standard error (SE). Bars with different letters are significantly different (*P* < 0.05) according to the LSD test.

The vase life of French marigold inoculated with a consortium of all three endophytic bacterial strains was 12 days compared to 6 days of control (Figure 2). Corresponding treatments of PsJN prolonged vase life by 10 days. Similarly, reduction in petal senescence was also significantly increased up to 8 days by MN17 and MN54. The inoculation with PsJN and consortia reported an increase in flower diameter up to 42.31 and 45.33 mm, respectively, compared to control (25.67 mm; Figure 2).

### Photosynthetic parameters and SPAD index

**FIGURE 3:**
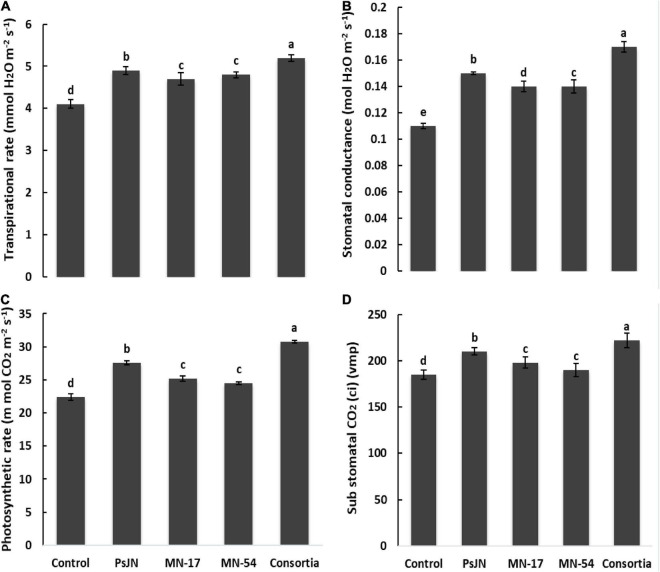
Effect of endophytic bacterial inoculation on **(A)** transpiration rate, **(B)** stomatal conductance, **(C)** photosynthetic rate, and **(D)** substomatal conductance of French marigold. Error bars represent the standard error (SE). Bars with different letters are significantly different (*P* < 0.05) according to the LSD test.

The chlorophyll contents in terms of SPAD value were higher in endophytic bacterial strains treated plants compared to the uninoculated control (Figure 1). A maximum SPAD value of 42.33 was observed in consortium treated plants, followed by strain MN17 (41.30) over the uninoculated control (24.30). The consortium application gave a maximum increase of up to 36.84% in photosynthetic rate compared to the control (Figure 3). Strain PsJN inoculation also reported a better increase in the photosynthetic rate of up to 23.21% compared to the uninoculated control. A maximum increase in transpiration rate of up to 26.82% was observed due to consortium application. The application of PsJN also showed a better transpiration rate with an increase of up to 19.51% over uninoculated control (Figure 3). The consortium application showed a significant increase of up to 54.54 and 23.41% in stomatal and sub-stomatal conductance, respectively, compared to the untreated control.

### Biochemical and antioxidant parameters

**FIGURE 4:**
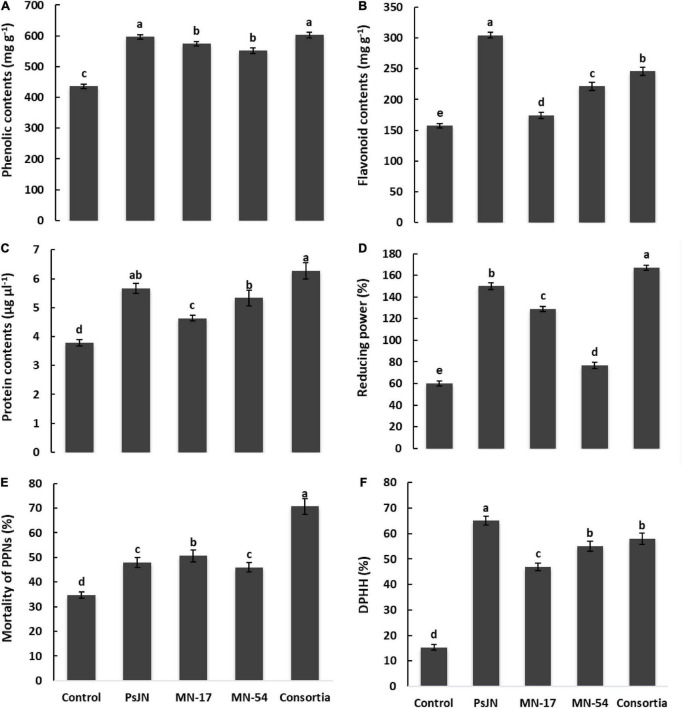
Effect of endophytic bacterial inoculation on **(A)** phenolic contents, **(B)** flavonoid contents, **(C)** protein contents, **(D)** ferric reducing power, **(E)** mortality of PPNs, and **(F)** DPPH radical scavenging activity of French marigold. Error bars represent the standard error (SE). Bars with different letters are significantly different (*P* < 0.05) according to the LSD test.

The increase in total phenolic and flavonoid contents was observed due to inoculation with endophytic bacterial strains (Figure 4). The consortium application reported maximum phenolic contents of 602.67 mg g^–1^ followed by strain PsJN (596.0 mg g^–1^) than the uninoculated control (435.67 mg g^–1^; Figure 4). A maximum increase in total flavonoid contents of 304.30 mg g^–1^ was recorded in strain PsJN treated plants compared to the untreated control (157.32 mg g^–1^). The maximum protein contents of 6.27 μg ml^–1^ were observed in consortium treated plants, followed by PsJN inoculated plants which reported 5.67 μg ml^–1^ of total protein contents compared to the control having 3.78 μg ml^–1^ of total proteins contents (Figure 4).

The positive effect of tested endophytic strains and their consortium application on ferric reducing power of French marigold flower extract had been demonstrated in Figure 4. Strain PsJN treated plants reported a higher ferric reducing ability of 150%, which was further extended up to 167% in consortium treated plants compared to the uninoculated control having a ferric reducing power of 60% (Figure 3). Strains MN17 and MN54 also showed a better ferric reducing ability of 129 and 77%, respectively, compared to the uninoculated control. Inoculation with endophytic bacterial strains reported increased DPPH scavenging activity of French marigold flower extract compared to the uninoculated control (Figure 4). The consortium application reported higher DPPH scavenging activity of 58% than the uninoculated control of 15% DPPH scavenging activity.

**FIGURE 5:**
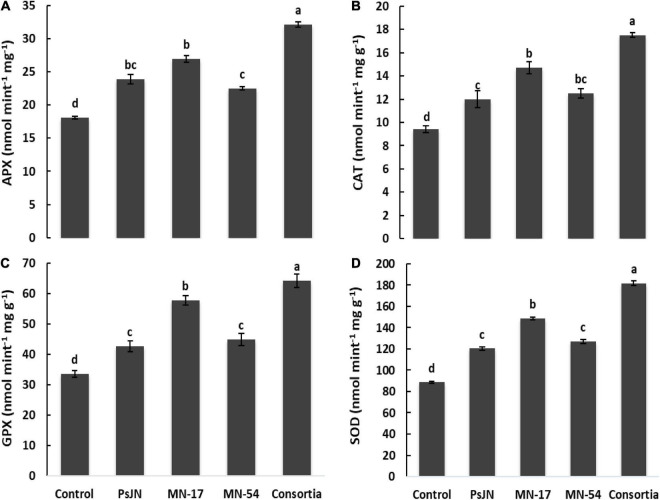
Effect of endophytic bacterial inoculation on **(A)** ascorbate peroxidase (APX), **(B)** catalase (CAT), **(C)** glutathione peroxidase (GPX), and **(D)** superoxide dismutase (SOD) activity of French marigold. Error bars represent the standard error (SE). Bars with different letters are significantly different (*P* < 0.05) according to the LSD test.

The increase in antioxidant activities was observed due to endophytic bacterial strain over control treatment (Figure 5). Strain PsJN, MN17, and MN54 increased the ascorbate peroxidase (APX) to 31.9, 48.6, and 24.6%, respectively. However, consortium application increased the APX up to 77.3% over treatment set as control. Similarly, strain MN17 increased the catalase (CAT), glutathione peroxidase (GPX), and superoxide dismutase (SOD) up to 48.6, 60.2, and 65.8%, respectively. However, combined application of endophytic bacterial strains increased the CAT, GPX, and SOD activity to 86.0, 91.6, and 102.9%%, respectively, over control treatment.

### Nematicidal and hemolytic activities

**FIGURE 6:**
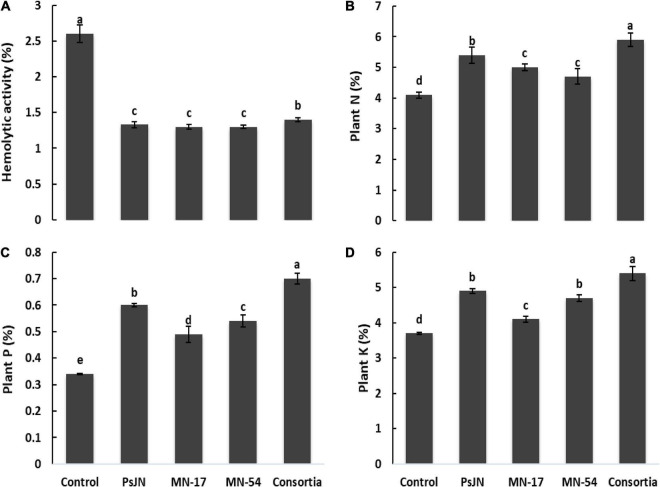
Effect of endophytic bacterial inoculation on **(A)** hemolytic activity, **(B)** plant N (%), **(C)** plant P (%), and **(D)** plant K (%) in French marigold. Error bars represent the standard error (SE). Bars with different letters are significantly different (*P* < 0.05) according to the LSD test.

French marigold flower extract from endophytic bacterial strains treated plants reported nematicidal activity against *Meloidogyne incognita* (Figure 4). The maximum nematicidal activity of up to 70.7% was recorded in consortium treatment, followed by strain MN17 which reported 50.7% higher nematicidal activity than uninoculated control. French flower extract of endophytic bacterial strains treated plants causes a reduction in hemolytic activity compared to the uninoculated control (Figure 6). A maximum decrease of 1.33, 1.30, and 1.30% in hemolytic activity of French flower extract was reported by strains PsJN, MN17, and MN54, respectively, over the uninoculated control of 2.6% hemolytic activity.

### Plant mineral contents

The N, P, and K contents in a shoot of French marigold were significantly promoted by inoculation with endophytic bacterial strains compared to the uninoculated control (Figure 6). The maximum increase of up to 43.9% in shoot N contents was observed in consortium treated plants followed by strain PsJN, which reported 37% higher shoot N contents compared to the uninoculated control. The maximum increase of up to 105.9 and 45.9% in shoot P and K contents, respectively, were obtained due to consortium application compared to the uninoculated control (Figure 6). Strain PsJN also reported a better increase of up to 60 and 33.3% in shoot P and K contents, respectively, over the uninoculated control.

### Enumeration of endophytic bacteria in the rhizosphere, root, shoot, and flowers

**FIGURE 7:**
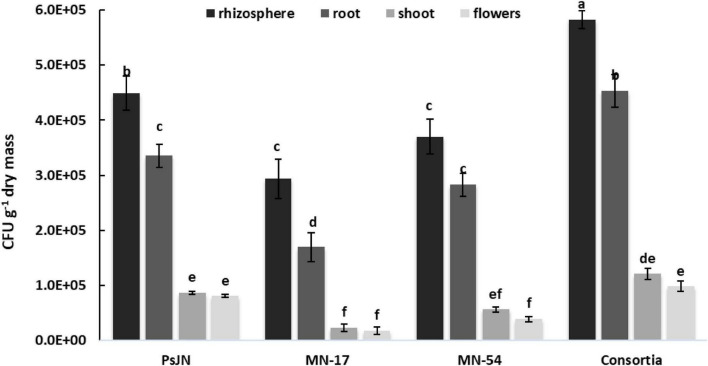
Persistence of selected endophytic strains in the rhizosphere, root, shoot, leaves, and flowers of French marigold. Error bars represent the standard error (SE). Bars with different letters are significantly different (*P* < 0.05) according to the LSD test.

Effective colonization of the applied strains was observed in the rhizosphere, root/shoot/leaves/flowers interior of French marigold (Figure 7). However, when used as a consortium, the persistence of selected strains was more enhanced relative to individual inoculation in the rhizosphere and tissue interior of French marigold plants. Inoculation with PsJN showed 4.49 × 10^5^ CFU g^–1^ rhizosphere, 3.36 × 10^5^ CFU g^–1^ root interior, 8.64 × 10^4^ CFU g^–1^ shoot interior, and 8.04 × 10^4^ CFU g^–1^ flowers bacterial population. However, the highest CFU g^–1^ dry weight of the inoculant strains was recovered from the rhizosphere (5.83 × 10^5^), root (4.53 × 10^5^), shoot (1.21 × 10^5^), and flowers (9.80 × 10^4^) in the consortium treatment.

### Pearson correlation and principal component analysis

**FIGURE 8:**
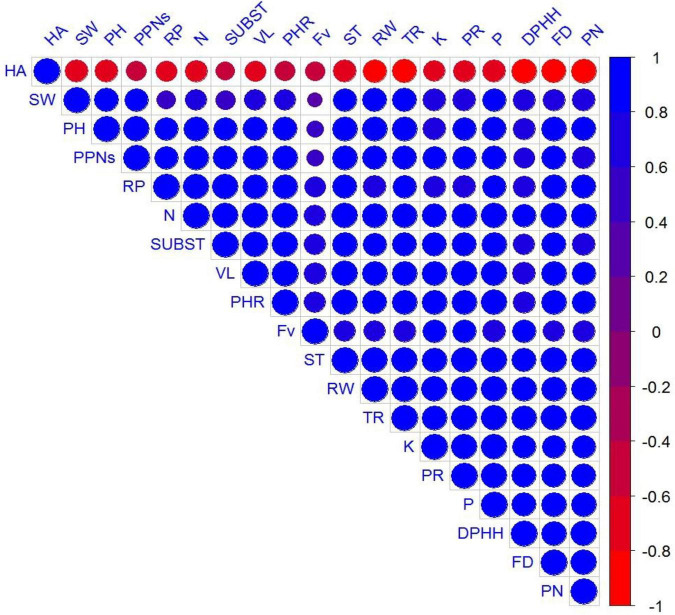
Represents correlation among measured parameters, where abbreviations of the correlation matrix are plant height (PH), shoot weight (SW), root weight (RW), flower diameter (FD), total flavonoids contents (FV), phenolic contents (PN), protein contents (PR), DPHH scavenging activity (DPHH), hemolytic activity (HA), vase life (VL), reducing power (RP), mortality of PPNs (PPNs), transpiration rate (TR), photosynthetic rate (PHR), stomatal conductance (ST), sub stomatal conductance (SUBST), plant N (N), plant P (P), and plant K (K).

A significant positive correlation was observed in growth attributes, vase life, biochemical parameters, physiological parameters, biological activities, and plant mineral contents (Figure 8).

**FIGURE 9:**
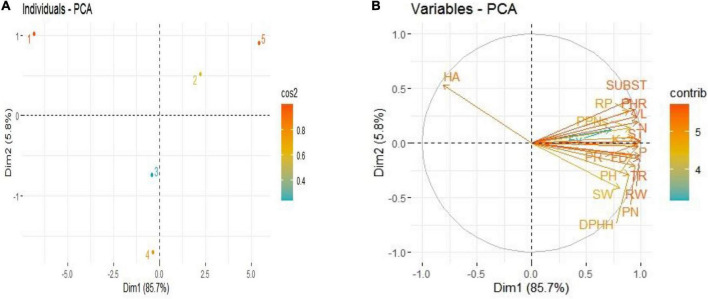
Score plots **(A)** and loading plots **(B)** of PCA on different attributes of French marigold by application of Endophytic bacteria alone and in combination. Score plots **(A)** represent the separation of treatments as (1) Control, (2) PsJN inoculation, (3) MN 17, (4) MN 54, and (5) Consortia. The abbreviations of loading plots **(B)** are plant height (PH), shoot weight (SW), root weight (RW), flower diameter (FD), total flavonoids contents (FV), phenolic contents (PN), protein contents (PR), DPHH scavenging activity (DPHH), hemolytic activity (HA), vase life (VL), reducing power (RP), mortality of PPNs (PPNs), transpiration rate (TR), photosynthetic rate (PHR), stomatal conductance (ST), sub stomatal conductance (SUBST), plant N (N), plant P (P), and plant K (K).

Principal component analysis (PCA) revealed interrelationships between different variables. The score and loading plots of PCA on some crucial traits of French marigold are shown in Figures 9A,B. Among all the components, the first two components *viz*. PC1 (Dim1) and PC2 (Dim2) exhibited maximum contribution and accounted for 91.5% of the total dataset. Of which PC1 contributed 85.7% while PC2 contributed 5.8%, respectively. All applied treatments were successfully separated by the first two principal components (Figure 9A). The distribution of the treatments gave a clear indication that endophytic bacterial strains sole or their consortium application had significant effects on studied attributes of French marigold compared to control. The sole application of endophytic bacterial strains was displaced more from control, and the consortium treatment was more displaced from control, indicating that it has a more pronounced effect. The first group of variables with which PC 1 is positively correlated includes plant height (PH), shoot weight (SW), root weight (RW), flower diameter (FD), total flavonoids contents (FV), phenolic contents (PN), protein contents (PR), DPHH scavenging activity (DPHH), hemolytic activity (HA), vase life (VL), reducing power (RP), mortality of PPNs (PPNs) transpiration rate (TR), photosynthetic rate (PHR), stomatal conductance (ST), sub stomatal conductance (SUBST), plant N (N), plant P (P), and plant K (K).

## Discussion

Chemical inhibitors such as silver thiosulphate (Veen and van de Geijn, 1978; Hyde et al., 2020), cyclic olefin norbornadiene (Reid and Wu, 1992; Depaepe and Van Der Straeten, 2020), and L-a-(aminoethoxyvinyl)-glycine (Nayani et al., 1998; Yu et al., 2022) are applied to inhibit plant ethylene production and to promote vase life of ethylene-sensitive flowers. However, the use of different chemicals has a variety of drawbacks associated with their application rate and method and high costs. Applying bio-effectors containing beneficial microorganisms instead of synthetic chemicals improves plant growth by supplying plant nutrients and may sustain environmental health and soil productivity. In the present study, three promising bioinoculants, including *B*. *phytofirmans* strain PsJN, *Enterobacter* sp. strain MN17, and *Bacillus* sp. strain MN54, were applied to French marigold seeds in the forms of sole inoculation and their consortium treatments. The treated plants were grown in a pot trial to investigate the effects of endophytic bacterial strains on plant attributes in terms of vase life, physiological, biochemical, antioxidant, and biological activities. By adopting the current biotechnological approach, we hypothesized that bioinoculants could competently dwell in the French marigold rhizosphere and improve the quality of their flowers in terms of their growth, vase life, chemical constituents, physiological, and biological activities (Dal Cortivo et al., 2020).

### Endophytes improve the growth attributes of French marigold plants

To sustain ornamental crop production under limited available resources, the greenhouse growers need to reduce the cost of production in terms of chemical fertilizers without losing crop quality. PGPR can improve plant growth in a resource-poor environment by improving plant nutrient availability. In the current study, the application of endophytic bacterial strains in sole inoculation and/or consortium treatment enhances the plant growth of French marigold in terms of plant height, root length, shoot weight, and root weight (Figure 1). The French marigold plants treated with a consortium of strains *B*. *phytofirmans* (PsJN), *Enterobacter* sp. (MN17), and *Bacillus* sp. (MN54) showed maximum increase in plant growth attributes compared to sole inoculation that may be due to accumulative and synergistic effect of endophytic bacterial strains that boosted the plant growth through involving their direct and indirect impact (Doty et al., 2009; Suherman and Anggoro, 2011; El-Deeb et al., 2012; Mustafa et al., 2019; Aziz et al., 2020; Nordstedt and Jones, 2020; South et al., 2021). These strains showed their potential *in vitro* plant growth-promoting (PGP) attributes, including production of indole-3-acetic acid and siderophore, solubilization of nutrients, and nitrogen fixation, which our research group previously reported (Samreen et al., 2019; Naveed et al., 2020a; Sabir et al., 2020). Similarly, Puri et al. (2020) said that *Caballeronia sordidicola* isolated from spruce seedlings in low-fertility soil possess PGP attributes and promoted plant growth attributes of spruce and pine tree seedlings. In this study, the increase in plant growth of French marigold due to the application of a consortium of endophytic bacterial strains might also be due to the production of phytohormones, including auxins, gibberellins, ethylene, cytokinin, and abscisic acid that can stimulate plant growth as chemical messengers. These hormones are essential in regulating plant growth and development in plants by regulating the process of organogenesis, cell division, expansion, and differentiation (Ryu and Patten, 2008).

### Endophytes improve physiological attributes of French marigold plants

In this study, the application of endophytic bacterial strains promoted plant physiological attributes, including chlorophyll contents, transpiration rate, photosynthetic rate, and stomatal and sub-stomatal conductance compared to the uninoculated control (Figures 1, 3). The beneficial influence of endophytic bacterial strains was more dominant in consortium treatment than in sole inoculation of strains. These beneficial effects of plant-bacteria interaction were previously reported by various researchers under numerous environmental stresses, including drought (Naveed et al., 2014), salinity (Ahmad et al., 2014), heavy metals (Naveed et al., 2020a,b), and hydrocarbon toxicity (Ali et al., 2020). Increased physiological attributes due to bacterial inoculation improved plant growth and biomass production of French marigolds. The increase in chlorophyll content due to a consortium of endophytic strains could be related to the established factor of higher enzymatic activities such as catalase and peroxidase (Singh, 1988; Kavino et al., 2010). The plant-associated bacteria logically boosted enzymatic activity due to their abundant biomass, higher metabolic activity, and extracellular enzyme production. They are also involved in plant defense by eliciting multiple antioxidant enzymes and counteracting oxidative stress by reactive oxygen species (Prasanna et al., 2013, 2017; Ibiang et al., 2017). The consortium of endophytic strains could be involved in regulating the transpiration of water and penetration of CO_2_ into the leaf by maintaining optimum moisture content. Such physiological phenomena could be helpful in rainfed cultivation by closing stoma and reducing water loss by preventing transpiration.

The application of PGPR showed their great prospect for improving nutrient availability and reducing the excessive application of chemical fertilizer. In the current study, inoculation with endophytic bacterial strains improves N, P, and K concentration in a shoot of marigold flower plants (Figure 5). The increase in nutrient concentration was more prominent in consortium treatment as compared to the sole application of endophytic bacterial strains. This increase in nutrient concentration could be due to the ability of endophytic bacterial strains to increase the bioavailability of nutrients in the soil and to improve its uptake and accumulation. Previously, we have reported the tested strains *viz*. *B*. *phytofirmans* (PsJN), *Enterobacter* sp. (MN17), and *Bacillus* sp. (MN54) *in vitro* ability to solubilize nutrients, especially P might improve its bioavailability in soil and enhance uptake and accumulation in plants shoot (Samreen et al., 2019; Naveed et al., 2020a; Sabir et al., 2020). Such nutrient solubilizing bacterial strains might also have the ability to solubilize other nutrients, including K and zinc, which can be possible in the current study as we have observed the increase in K concentration in plants of the marigold flower. Our results are similar to the findings of Khanghahi et al. (2018), Mumtaz et al. (2018, 2020), and Ahmad et al. (2019a). Such nutrients solubilizing bacteria adopted a variety of mechanisms, including acidolysis, reduction in pH, enzymolysis, and complexation through extracellular polysaccharides (Welch et al., 1999; Parmar and Sindhu, 2013). The mechanisms of association of plant and endophytic bacterial strains are not precise for mineral solubilization that might result from the secretion of organic acids of ligands specific to elements (Duangpaeng et al., 2012). Such ligands can alter substrate pH and enhance insoluble compounds’ chelation (Forchetti et al., 2010; Khan et al., 2020). The free-living rhizosphere and endophytic bacteria also demonstrated N-fixation in roots of nonleguminous crops (Nag et al., 2020) which might be valid for the current endophytic bacteria strains that showed an increase in N concentration in marigold flower plants. The precise mechanism of association of free-living endophytic bacteria with plant species is not well-known. It may be due to the presence of plant-specific root exudates that recruits plant-available bacteria and carry the microbiota from one generation to another (Toju et al., 2018; Van Deynze et al., 2018; Eyre et al., 2019).

### Endophytes improve the vase life of French marigold flowers

Bacteria associated with plants improve the quality of their flowers. In the present study, the uninoculated control revealed the lowest flower quality in terms of their numbers, diameters, and vase life (Figures 1, 2) which might be due to higher ethylene production during the flower ripening process. Higher ethylene production at ripening time causes several stress changes, including rapid loss of chlorophyll, proteolysis, loss of catalase activity, and increased membrane permeability (Ranwala and Miller, 2005). However, the increase in these flower’s quality attributes of marigold was observed due to the sole and/or consortium inoculation with endophytic bacterial strains, which might be due to the ability of these bacteria to produce ACC-deaminase that reduces the ethylene level in plants and delay flower ripening (Moon and Ali, 2022). This ethylene reduction in plants by ACC-deaminase-producing bacteria is a critical property that enables interference with the physiological processes of the host plant (Eun et al., 2019; Ali et al., 2022). Various commercially available chemicals such as potassium permanganate, ultraviolet lamps, activated charcoal, and catalytic oxidizers inhibit the ethylene concentration in fruits and flowers to improve vase life (Ebrahimi et al., 2021). However, improving the vase life of flowers through applying rhizosphere and endophytic bacteria is a novel biotechnological approach that is quite efficient in increasing flower quality and composition.

### Endophytes improve biochemical and antioxidant attributes of flowers extract

We reported the increase in the total content of phenolics, flavonoids, and protein in French marigold flower extract due to inoculation with endophytic bacterial strain (Figure 4). The consortium of endophytes mediated responses in French marigold flower extract promoted these metabolites in flower extract that played a cumulative, synergistic role in the enhancement of plant growth and flower quality. Previously, various researchers showed evidence of total proteins, phenolics, and flavonoid contents in plant-microbe interaction (Weir et al., 2004; Brencic and Winans, 2005; Bhattacharya et al., 2010). The flavonoid and phenolic contents in the extract of French marigolds could quench free radicals and act as antioxidants involved in anti-inflammatory and anti-carcinogenic responses (Biglari et al., 2008). We observed higher flavonoid and phenolic contents in marigold flower extract due to the consortium application of endophytic bacterial strains that could manifest higher antioxidant capacities. Further, consortium application also reported a higher increase in antioxidant activities in terms of DPPH radical scavenging, APX, CAT, GPX, and SOD activity and ferric reducing power of extract from French marigold flowers (Figure 4). Li et al. (2007) analyzed the 11 Chinese cultivars of marigold flowers extracted with ethanol, ethyl acetate, and n-hexane through high-performance liquid chromatography-mass spectrometry. They reported marked variation in total phenols, flavonoids, antioxidants, and radical-scavenging activities in the tested marigold cultivars.

Similarly, Duan et al. (2007) and Liu et al. (2009) reported polyphenols extracted from lychee-fruit pericarp to possess the scavenging activity against DPPH free radical, superoxide anions, and hydroxyl radicals. Additionally, a reduction in hemolytic activity was observed in the current study due to inoculation with sole and demonstrating the ability of endophytic bacterial strains to reduce the hemolytic activity of French marigold flower extract. The reduction in hemolytic activity was more in sole inoculation than in a consortium which might be due to the application of a single organism in sole inoculation with endophytic bacteria. The reduction in hemolytic activity due to inoculation can be due to the increased production of antioxidants because of bacterial application in French marigold plants.

### Endophytes improve the nematicidal activity of French marigold flowers extract

Some marigold varieties are resistant to PPNs due to their allelopathic potential (Wang et al., 2007; Hooks et al., 2010). A blue-fluorescing compound called α-terthienyl in marigold plants was recognized for its allelopathic potential against nematodes, insects, fungi, and viruses and cytotoxic activities (Zechmeister and Sease, 1947; Wang et al., 2007). In this study, endophytic bacterial strains promoted the nematicidal activity of extract from French marigold flowers compared to the uninoculated control (Figure 5). This increase in nematicidal activity could be due to the role of endophytic bacterial strains in enhancing the production of nematicidal phytocompounds specially α-terthienyl. Various rhizospheric and endophytic bacterial strains are also well-known for their potential role in the biocontrol of phytopathogen and are involved in indirect plant growth promotion (Mhatre et al., 2019; Gamalero and Glick, 2020), which might be true for current endophytic bacterial strains. Sturz and Kimpinski (2004) isolated several bacterial endophytes from African and French marigolds that showed their nematicidal activities against *Pratylenchus penetrans* in soils. Such plant bacterization could be a potential candidate for non-residual and environment-friendly pesticides that will boost the plant’s defense metabolites to reduce the pathogens population in the root zone (Nivsarkar et al., 2001).

## Materials and methods

### Preparation of endophytic bacterial inoculum

Three pre-isolated endophytic PGP bacterial strains, *viz*. *B. phytofirmans* PsJN (Naveed et al., 2020a), *Enterobacter* sp. MN17 (Sabir et al., 2020), and *Bacillus* sp. MN54 (Samreen et al., 2019) were collected from Environmental Science Laboratory, Institute of Soil and Environmental Sciences (ISES), University of Agriculture Faisalabad (UAF), Pakistan. These strains were grown separately in Luria-Bertani (LB) broth containing tryptone (10 g L^–1^), yeast extract (5 g L^–1^), and NaCl (10 g L^–1^) at 28 ± 1°C and 100 rpm for 48 h in an orbital shaking incubator (Firstek Scientific, Japan). The optical density (OD) at 600 nm of each broth was adjusted to 0.5 using a spectrophotometer (Gene Quant Pro, Gemini BV, Netherlands) to obtain a uniform population of bacteria [10^8^–10^9^ colony forming units (CFU) ml^–1^].

### Seed bacterization

The peat as a carrier material was sterilized at a pressure of 138 kPa and temperature of 121°C for 30 min and inoculated with bacterial broth culture. The peat-based inoculum was incubated at 28 ± 2°C by adding a 10% sugar solution to increase the microbial populations. For inoculation, the desired suspension of inoculum (10^8^–10^9^ CFU ml^–1^; 250 ml kg^–1^ peat) was mixed with sterilized peat and incubated for 24 h at 28 ± 2°C before use for seed coating (seed to peat ratio 1.25:1 w/w). Marigold seed dressing was prepared with the inoculated peat mixed with 10% sterilized sugar (sucrose) solution in a 10:1 ratio (Saeed et al., 2019). In the case of un-inoculated control, seeds were coated with the sterilized peat treated with broth, and 10 % fixed sugar solution.

### Pot experiment and treatment plan

A pot experiment was conducted in the net-house, ISES, UAF, Pakistan, to evaluate the potential of endophytic bacteria to improve flowering, pharmacological activities, and delay of flower senescence of French marigold. The soil used for the experiment was collected from the field, air dried, thoroughly mixed, passed through a 2-mm sieve, and analyzed for various physical and chemical characteristics. The soil was sandy clay loam, having pH, 7.88; EC, 1.38 dS m^–1^; organic matter, 0.78%; total nitrogen, 0.034%; available phosphorus, 7.80 mg kg^–1^ and extractable potassium, 117 mg kg^–1^. French marigold seeds were surface sterilized by dipping in 70% ethanol for 2 min and treated with 1.5% NaClO for 5 min, followed by washing three times with sterile distilled water (1 min each time). The efficacy of surface sterilization was checked by culturing seeds and aliquots of the final rinse in LB agar plates where no growth was observed. The experiment contained the following treatments: (1) Control, (2) PsJN inoculation, (3) MN-17 inoculation, (4) MN-54 inoculation, and (5) Consortia of PsJN, MN-17, and MN-54. Surface-disinfected marigold seeds were coated with different endophytic bacterial treated slurry. In the control treatment, slurry for seed coating was prepared using sterilized LB broth. Pots were arranged in the net-house using a completely randomized design with three replications of each treatment. The plants were harvested after 60 days during full bloom flowering and further processed immediately for growth, physiological, and pharmacological analysis.

### Assessment of growth parameters and vase life

The end of vase life was determined as the time in which 50% of open flower petals had wilted. The vase life of French marigold flowers was recorded by the number of days from the day that cut flowers were put in distilled water and incubated at room temperature (24°C for 12 days) until they had no ornamental value (underwent a color change, wilt, or loose turgidity). The mean value of the vase life of all flowers in each was calculated as the average vase life for each treatment. Plant agronomic parameters such as plant height, shoot, root fresh weight, root length, number of flowers plant^–1^, and flower diameter were recorded after harvesting the French marigold plants.

### Physiological parameters

Plant physiological parameters of both treated and untreated plants were recorded at mid-day (between 10:00 and 14:00). A portable infrared gas analyzer [IRGA (CI-340) Germany] was used (at 1,200–1,400 μmol m^–2^ s^–1^ photosynthetic photon flux density) to measure transpiration rate, photosynthetic rate, stomatal, and substomatal conductance. Relative chlorophyll contents SPAD index of the second leaf from apex were recorded through Chlorophyll meter at vegetative stage.

### Estimation of total flavonoid, phenolic acid, and protein contents

To determine total flavonoid contents, 0.5 ml of French marigold flowers extract was mixed with 2 ml of distilled water and 0.15 ml of NaNO_2_ (5%) solution and incubated for 6 min. After that, 0.15 ml of 10%, AlCl_3_ solution was added and incubated for 6 min, followed by adding NaOH (4%) solution to the mixture. The volume of the reaction mixture was made up to 5 ml by adding methanol. The absorbance of the reaction mixture was taken at 510 nm after incubation for 15 min. Total flavonoid contents of the extracts were expressed as catechin equivalents from the linear regression curve of catechin (Park et al., 2008). The total phenolic compounds in French marigold flowers extract were determined by the Folin–Ciocalteu method (Bärloche and Graça, 2020). The calibration curve was prepared with different concentrations of gallic acid. To estimate total protein contents, 0.1 ml of French marigold flower extract and 0.1 ml of NaOH (2N) were mixed and hydrolyzed at 100°C. The freshly prepared complex-forming reagent (1 ml) consisting of Na_2_CO_3_ (2%), CuSO_4_.5H_2_O (1%), and KNAC_4_H_4_O_6_.4H_2_O (2%) was added to cooled hydrolysate (Lowry et al., 1951). After 10 min of incubation, 0.1 ml of Folin reagent was added through a vortex and incubated for 30 min. Total protein contents were estimated by taking absorbance at 750 nm (Lowry et al., 1951).

### DPPH radical scavenging and antioxidant power assays

The antioxidant activity of French marigold extract was estimated through DPPH radical scavenging assay as performed by Yen and Chen (1995) with slight modifications. The freshly prepared 1 ml of DPPH solution was added to 3 ml of marigold flower extracts at different concentrations and kept for 30 min in the dark. After incubation, absorbance was noted at 517 nm. A low absorbance of the reaction mixture indicates a high radical scavenging activity. The antioxidant activity of butylated hydroxytoluene and ascorbic acid was analyzed as standards. The solution without marigold flower extract was used as a control. The inhibition of DPPH radical samples was calculated as follows.


(1) $$DPPHinhibition\left(\%\right)=\left[AC-\frac{A\,S}{A\,C}\right]\times 100$$

where, AC, absorbance of control; AS, absorbance of sample.

The ferric reducing power of French marigold flower extract that reflected their antioxidant activity was determined using Fe^3+^ and Fe^2+^ reduction assay (Benzie and Strains, 1996). The 1 ml of French marigold flowers extract in methanol was added to 2.5 ml of sodium phosphate buffer (0.2 M; PBS) and 2.5 ml of K_3_Fe(CN)_6_ (1%) solution. The solution was incubated at 50°C for 20 min on a vortex shaker, followed by adding 2.5 ml of trichloroacetic acid (10%). The final volume of 2.5 ml after centrifugation was diluted up to 5.0 ml with deionized water, and absorbance was read at 700 nm.

For antioxidant enzyme determination, frozen leaf material was homogenized in potassium phosphate buffer (0.2 M, pH 7) ice-cold solution with ethylene diamine tetra acetic acid (EDTA) (0.1 mM). The activity of APX was measured by a decline in spectrophotometer absorbance (290 nm wavelength) due to the reduction of ascorbate by H_2_O_2_ (Nakano and Asada, 1981). The activity of CAT enzymes was observed by a diminution in the spectrophotometer absorbance (240 nm wavelength) owing to H_2_O_2_ loss (Cakmak and Marschner, 1992). The activity of GPX was observed by spectrophotometric absorbance (340 nm wavelength) due to the reaction of a sodium azide, glutathione, and GPX solution into a β-NADPH (nicotinamide adenine dinucleotide phosphate) (Aebi, 1983). The activity of SOD enzyme was estimated by spectrophotometric absorbance (420 nm wavelength) due to reaction of enzyme extract with sodium phosphate, EDTA and pyrogallol (Roth and Gilbert, 1984).

### Hemolytic and nematicidal activities

Hemolytic activity of French marigold extracts was assayed through the method of Powell et al. (2000). The human blood cells (3 ml) were gently poured into a sterile falcon tube and washed three times with chilled PBS (5 ml) by centrifuging the tubes for 5 min. The red blood cells (180 μl) were gently mixed with French marigold flower extract (20 μl) and centrifuged for 5 min. The supernatant (100 μl) was diluted with chilled sterile PBS (900 μl). Triton X-100 was run as a positive control, and PBS was taken as a negative control in triplicate. Absorbance was taken at 576 nm through ELISA plate reader.

The nematicidal activity of endophytic bacterial strain treated French marigold flowers extract was evaluated. A root-knot nematode *M. incognita* at juveniles J2 stage was obtained by incubating nematode egg masses in tap water at 27°C in the dark. They were collected every 2 days and concentrated in small volumes of sterilized water by filtering through 1 μm Whatman filters and collecting them after repeated washes (Molinari, 2009). The soil stages juveniles of nematodes were separately transferred to oil solutions from French marigold flower extract in Petri dishes. Each treatment had 100 nematodes in triplicate. Nematodes in distilled water and 0.05% Tween solutions were served as checks. The Petri dishes were kept at room temperature (28 ± 2°C). Numbers of un-hatched and *M. incognita* juveniles were daily recorded for 16 days, and immobile soil stages of nematode were counted after 24 and 72 h (Abd-Elgawad and Omer, 1995). Each time, the nematodes were transferred in aerated distilled water, and then the active nematodes were counted after a day.

### Plant analysis

Post-harvest plant biomass (above and below ground) was obtained after drying whole plants at 65°C for 72 h, and samples were wet digested by following the method of Wolf (1982). The plant digested samples were analyzed for N, P, and K concentration by following the technique of Estefan et al. (2013). The N contents in plant digest were determined through the Kjeldahl method. The P concentration was estimated through a colorimetric method, while the K concentration was determined using a flame photometer.

### Persistence of endophytic bacteria in the rhizosphere, root, shoot, and flowers

The rhizosphere and endophytic persistence of selected bacterial strains were determined by dilution and plate counting technique. For colonization assay, rhizospheric soil was collected, and soil slurry was prepared at a ratio of 1:5 (soil: NaCl 0.9%) following agitation for 30 min at room temperature. After sedimentation, serial dilutions up to 10^–6^ were plated onto a 10% tryptic soy agar medium. Colonies were counted after incubating the plates at 28 ± 1°C for 2-days, and the colonization value was determined following the equation.


(2) $$N\,o\,o\,f\,c\,o\,l\,o\,n\,i\,e\,s\,\left(C\,F\,U\,g^{-1}\,d\,r\,y\,w\,e\,i\,g\,h\,t\right)=1/d\,i\,l\,u\,t\,i\,o\,n\,f\,a\,c\,t\,o\,r\times numberofcolonies/dryweight$$

Similarly, 1 g of surface-sterilized samples of each plant tissue (root, shoot, leaves, and flowers) were homogenized in 5 ml 0.9% saline buffer using a sterile mortar and pestle. After settling the solid fraction, serial dilutions up to 10^–5^ were spread on a TSA medium. Twenty-five visible colonies were selected per treatment randomly, and their identity with that of inoculant strain was confirmed by restriction fragment length polymorphism (RFLP) analysis of the 16S–23S rRNA intergenic spacer (IGS) region (Naveed et al., 2014).

### Statistical analysis

Data for different growth and yield attributes were collected and analyzed statistically using software “Statistix 8.1^®^” version. Means were compared by using least significant difference (LSD) test (Steel et al., 1997) at a 5% probability level. Origin Pro 9.1 software was used for graphs, and Pearson correlation, PCA was performed using R-software.

## Conclusion

The current study concluded that French marigold plants inoculated with endophytic bacteria improve plant growth, physiology, nutrient uptake, and vase life compared to the uninoculated control. They also improve the metabolites, antioxidant enzyme activity, and nematicidal activities of extract from French marigold flowers. The application of endophytic bacteria is also involved in reducing the hemolytic activity of extract from French marigold flowers. The consortium application reported a significantly more significant improvement in French marigold attributes than sole inoculation. Consortium application of such compatible promising endophytic bacteria could benefit the horticulture industry by providing evidence that beneficial bacteria adopted as an effective tool to reduce fertilizers input and improve plant metabolites profile and pharmaceutical quality of ornamental plants.

## Data availability statement

The original contributions presented in the study are included in the article/supplementary material, further inquiries can be directed to the corresponding authors.

## Author contributions

MN, AM, and MB: conceptualization. SH and MR: methodology and formal analysis. SH, MR, MM, and ZS: software. MN, OM, TH, and JH: validation. MN, MB, AK, and JH: resources. SH, ZS, and MN: data curation. SH, MN, and MR: writing—original draft preparation. MN and AM: writing—review and editing. TH, MB, JH, and AM: supervision. MN: project administration. AK, JH, AM, MB, and MN: funding acquisition. All authors have read and agreed to the published version of the manuscript.
